# Quiescence Through the Prism of Evolution

**DOI:** 10.3389/fcell.2021.745069

**Published:** 2021-10-29

**Authors:** Bertrand Daignan-Fornier, Damien Laporte, Isabelle Sagot

**Affiliations:** Univ. Bordeaux, CNRS, IBGC, UMR 5095, Bordeaux, France

**Keywords:** quiescence, multicellularity, evolution, unicellular organism, microenvironment

## Abstract

Being able to reproduce and survive is fundamental to all forms of life. In primitive unicellular organisms, the emergence of quiescence as a reversible proliferation arrest has most likely improved cell survival under unfavorable environmental conditions. During evolution, with the repeated appearances of multicellularity, several aspects of unicellular quiescence were conserved while new quiescent cell intrinsic abilities arose. We propose that the formation of a microenvironment by neighboring cells has allowed disconnecting quiescence from nutritional cues. In this new context, non-proliferative cells can stay metabolically active, potentially authorizing the emergence of new quiescent cell properties, and thereby favoring cell specialization. Through its co-evolution with cell specialization, quiescence may have been a key motor of the fascinating diversity of multicellular complexity.

## Quiescence in Unicellular Organisms

Life is characterized by the ability of self-reproduction. However, natural selection, which is key to Darwinian evolution, operates on inherited variations that increase individual’s ability not only to reproduce but also to survive. This duality started with the very first unicellular organisms for which there was a need for trade-off between proliferation and long-term survival in changing environments. In this early scenario, quiescence could have emerged as an adaptive reversible proliferation arrest. Quiescence, which is not strictly required for life to multiply, might have resulted from the need to control proliferation in response to unfavorable environmental conditions that otherwise would compromise viability by disconnecting cell division from cell growth. At this time, quiescence might have represented an extreme form of slow growth and been a passive consequence of a massive anabolism slow-down due to resource limitation. Similarly, in contemporary unicellular prokaryotes or eukaryotes, the cessation of cell cycle progression *per se* may still be just a rather passive outcome of a drastic anabolic diminution. This was proposed for *Saccharomyces cerevisiae* cells that were observed to cease to proliferate in various cell cycle phases (Brauer et al., 2008; Daignan-Fornier and Sagot, 2011a,b; Klosinska et al., 2011; Laporte et al., 2011; Broach, 2012) as well as for *Cryptococcus neoformans* (Takeo et al., 1995) and *Schizosaccharomyces pombe* (Costello et al., 1986; Wei et al., 1993).

Yet, in modern unicellular organisms, additional quiescent cell properties improving survival capacities have appeared. These new intrinsic abilities involve dedicated molecular processes that protect cells from adversities (Rittershaus et al., 2013; Zhang and Cao, 2017). For example, the cellular response for survival can engage the storage of various energetic macromolecules, the nature of which depends on both the limiting resource and the species (Montrose et al., 2020). This was demonstrated in *S. cerevisiae* and *Chlamydomonas reinhardtii*, in which the metabolic rewiring upon quiescence establishment is highly dependent on the nature of the exhausted nutrient (Klosinska et al., 2011; Broach, 2012; Takeuchi and Benning, 2019). In addition, quiescence establishment is often accompanied by the remodeling of organelles and the reorganization of cellular machineries. This has been particularly documented in quiescent *S. cerevisiae* in which multiple reorganizations were observed, including the hyper-condensation of the genome, the rearrangement of the mitochondrial network, or the aggregation of enzymes (O’Connell et al., 2012; Prouteau et al., 2017; Sagot and Laporte, 2019b; Miles et al., 2021). Some of these reorganizations are the consequences of variations in the cytoplasm physico-chemical properties occurring upon proliferation to quiescence transition (Joyner et al., 2016; Munder et al., 2016; Heimlicher et al., 2019). Others may be the outcome of a dedicated signaling pathway. The possible, yet not mandatory, physiological “raisons d’être” of many of these cellular reorganizations are still puzzling, but it has been speculated that the accumulation of reserves of macromolecules, such as osmoprotective polymers, or cellular machineries, such as ribosomes, may be a key for adaptation in a changing environment (Argüelles, 2000; Yu et al., 2020). Altogether, the diverse properties of quiescent cells, which probably have evolved in different ways in function of both the cell types and the environmental conditions, exemplify the plasticity of this cellular state (Sagot and Laporte, 2019a,b).

In parallel, it seems that unicellular organisms have evolved a fast and efficient quiescence exit. For example, *S. cerevisiae* responds immediately to an extracellular food supply (Zhang et al., 2019) and many of the cellular machineries reorganized upon quiescence establishment, such as the actin cytoskeleton or the proteasome, are mobilized within seconds upon carbon replenishment (Sagot et al., 2006; Laporte et al., 2008, 2011). Furthermore, during the first step of resuscitation from chlorosis of nitrogen starved cyanobacteria, an almost instantaneous increase in the adenosine triphosphate (ATP) level is observed upon addition of sodium nitrate (Neumann et al., 2021), just as in quiescent *S. cerevisiae* upon glucose re-feeding (Laporte et al., 2011). Besides, in *S. cerevisiae*, the polymerase II is poised onto promoter of genes that are critical for proliferation resumption (Radonjic et al., 2005). In parallel, the chromatin remodeling complex (RSC) is bound to genes induced upon quiescence exit and facilitates rapid gene expression firing, despite a globally repressive chromatin state maintained compacted by histone specific modifications (McKnight et al., 2015; Swygert et al., 2019; Cucinotta et al., 2021). Hence, unicellular quiescent cells seem to be prepared to respond rapidly to favorable conditions and mechanisms accompanying quiescence establishment may be an asset for the competition within a given environment. Of note, this propensity to swiftly exit quiescence has been proposed to be the Achilles’ heel of persisters pathogenic bacteria that can be deceived by specific metabolites that trigger quiescence exit and as such expose them to killing drugs (Amato et al., 2014; Prax and Bertram, 2014). Thus, in contemporary unicellular organisms, quiescence establishment involves reorganizations to improve both sustained protection and fitness upon quiescence exit.

Finally, in both prokaryotes and some unicellular eukaryotes, it has been observed that the longer the time spent in quiescence, the slower the resumption of proliferation, suggesting that quiescence might deepen with time by unknown mechanisms that remains to be deciphered (Su et al., 1996; Laporte et al., 2017; Pu et al., 2019).

Overall, the rationale governing quiescence reflects an opportunistic behavior that seems very well-adapted to inter-individual competition in a fluctuating environment. Yet, while in unicellular species, cell and organism are synonymous, in multicellular entities, the two scales are distinct. How did cellular quiescence evolve in this context?

## Quiescence in Rudimentary Multicellular Organisms

In *C. reinhardtii*, as in other unicellular algae (*Chlorella*, *Scenedesmus*), non-favorable environmental conditions induce not only a cell cycle arrest but also cell clumping into large aggregates made of few tens to thousand quiescent cells held together by an extracellular matrix. This multicellular form favors resistance to starvation, desiccation, and freezing. When conditions become favorable again, aggregates disassemble in few minutes and cells re-proliferate (Sathe and Durand, 2016; de Carpentier et al., 2019). In this case, quiescence is still a survival form, but it is associated with the formation of a dedicated multicellular assembly. *Dictyostelium discoideum* also propagates as a single cell in a nutrient rich environment, and upon nutrient exhaustion, just as other dictyostelids, it may opt for several strategies. Unicellular forms can just stop proliferating, forming a so-called solitary quiescent cells that survive short periods of starvation. Alternatively, a cell can encyst to form a unicellular quiescent microcyst that is able to survive long period of scarcity. Nutritional depletion, when combined with dark and humid environment can also lead to the initiation of a sexual cycle that will end up by the formation of a dormant macrocyst from a diploid giant cell. Finally, upon starvation, *D. discoideum* cells can also start to secrete both chemoattractant and extracellular matrix and aggregate into a multicellular sorogen, that can eventually form a stalk, named fruiting body, in which some cells differentiate into dormant spores that will disseminate and germinate when external conditions will become favorable again (Dubravcic et al., 2014; Kin and Schaap, 2021). Thus, in dictyostelids, cellular quiescence is still associated to survival in non-optimal environmental conditions, but combined with multicellularity, it contributes to improve propagation.

In the above examples, planktonic organisms can switch to a temporary multicellular lifestyle to improve survival. Symmetrically, volvocine green algae species, which live as spherical assembly of thousands of cells in rich freshwater habitats, can produce dormant unicellular zygotes capable of surviving adverse conditions. The return to a favorable environment triggers meiosis and haploid offspring reproduces asexually to ultimately rebuild a multicellular form (Hallmann, 2011). Similarly, many multicellular organisms use quiescent unicellular spore as a mean to both face unfavorable conditions and disseminate. These quiescent spores can be generated asexually, such as conidiospores produced by several kind of filamentous fungi like *Neurospora crassa* (Turian and Matikian, 1966; Ruger-Herreros and Corrochano, 2020), or *via* sexual reproduction like ascospores formed by ascomycetes (Bennett and Turgeon, 2016) or micro- and macro-spores generated by algae (Maggs and Callow, 2003). In these rudimentary multicellular species, quiescence is a combination between a survival mode and a mean to disseminate robust single-celled propagules.

## Quiescence in More Complex Multicellular Organisms

### Suspended Animation at the Whole Organism Scale

Where multicellularity has become obligatory, some selected survival strategies were no longer based on cellular quiescence but rather take place at the whole organism scale. In that situation, extended period of inactivity, either adaptive or programmed, where developed. Cryptobiosis is an extreme form of inactivity that can lead to an almost complete cessation of the body metabolism. It includes freezing, desiccation, hypoxia and osmobiosis. This state of “suspended animation” is observed in lichen or mosses, but also in invertebrate such as nematodes or tardigrades. It is also widely used by a variety of phytoplankton species that can persist for multiple decades in a quiescent resting state (Ellegaard and Ribeiro, 2018). This adaptive reaction allows surviving unpredictable unfavorable conditions. By contrast, organism dormancy is programmed and widely found in plants and animals. Dormancy can either be an obligated part of the life cycle, such as a given developmental stage like diapause, or a seasonal arrest that anticipates predictable environmental non-favorable conditions, such as hibernation (for an excellent review see Withers and Cooper, 2010). Yet, many multicellular species do not have recourse to suspended animation to survive.

### Key Aspects of Unicellular Quiescence Have Been Conserved in Multicellular Species

In multicellular organisms, cell death is no longer synonymous to organism death and within a multicellular body, dead cells are generally replaced to preserve tissue homeostasis. In adults, this replacement relies on stem cells that are capable of surviving and self-renewing all along the organism’s lifetime (Rumman et al., 2015). Quiescence favors stem cells preservation by preventing the accumulation of replication-induced mutations (Fuchs, 2009; Cheung and Rando, 2013; Tümpel and Rudolph, 2019). Quiescence also permits stem cells to have an inherent low metabolic activity. In particular, the low rate of oxygen consumption reduces the impact of deleterious oxidation accrual thereby limiting the damaging effect of age on cellular macromolecules. Furthermore, as in *S. pombe*, in stem cells, quiescence specific protective and repair mechanisms limit DNA damages, hence avoiding their propagation to daughter cells. Thus mechanisms implemented in quiescence are critical for both stem cell survival and fitness of the progeny (Mandal et al., 2011; Burkhalter et al., 2015; Gangloff and Arcangioli, 2017; Vitale et al., 2017). In this perspective, stem cell quiescence in multicellular organisms has a survival role quite similar to quiescence in unicellular species.

As observed in unicellular eukaryotes, quiescence establishment in cells of multicellular species is accompanied by the reorganization of cellular machineries. For example, quiescent primary human fibroblasts exhibit a tighter chromatin compaction (Evertts et al., 2013). The proteasome is reorganized into cytoplasmic granules in the root cells of *Arabidopsis thaliana* seedlings (Marshall and Vierstra, 2018) and the actin cytoskeleton form aggregates in non-dividing *Papaver rhoeas* pollen tubes, and rat endothelial cell (Jensen and Larsson, 2004; Poulter et al., 2010). However, to date, the cell biology of plant and animal quiescent cells remains largely underexplored.

Interestingly, quiescent cells from multicellular species can be paused in various cell cycle stages, just as some quiescent unicellular eukaryotes. This is the case of precursor cells of the *Drosophila melanogaster* wing discs that are arrested in G2 waiting for proliferation signals to form bristles (Nègre et al., 2003). Furthermore, an arrest in G2 has been associated with efficient adult stem cell regeneration in a variety of organisms, including hydra, axolotl, and zebrafish (Sutcu and Ricchetti, 2018). In addition, just as in yeast, it was observed that several quiescent stem cells are poised to possibly resume proliferation as fast as possible, an essential step for repair in various tissues (Cao et al., 2017; Relaix et al., 2021). Indeed, in muscle, systemic signals released upon muscle injury, prime some quiescent muscle stem cells into a pre-activated state by transitioning from a G0 state to a G alert state (Rodgers et al., 2014). Similarly, some neural stem cells become pre-activated for reentering the cell cycle more readily upon the next round of injury (Llorens-Bobadilla et al., 2015) and dormant hematopoietic stem cells differ in self-renewal potential and division frequency depending on their individual endogenous CDK6 level (Laurenti et al., 2015). Therefore, just as in unicellular organisms, quiescence exit swiftness is key, but in multicellular species, the trigger has shifted from nutrient cues to repair or renewing signals. Yet the logic stays the same, ensuring the flexibility for quiescent cell to respond to an ever-changing environment.

The co-existence of “deep” next to “alerted” quiescent stem cells indicated that for a given cell type, different kind of quiescent cells coexist within the same tissue, and pointed to an heterogeneity along the quiescence-to-reproliferation trajectory (Ancel et al., 2021). In fact, as in *S. cerevisiae*, differences in quiescence deepness were observed in mammals, in which quiescence exit become slower with age. For example, long-term quiescent hepatocytes (Roth and Adelman, 1974) or fibroblasts (Soprano, 1994; Kwon et al., 2017; Fujimaki et al., 2019) take a longer time to reenter the cell cycle than their younger counterparts and become less and less sensitive to proliferation stimulation. Finally, aged quiescent cells may ultimately transition to senescence, a non-proliferative cellular state that is irreversible (Sousa-Victor et al., 2014; Fujimaki and Yao, 2020). Thus, quiescence deepening reveals the limitation of cell replacement relying on stemness and is among the strongest candidates for multicellular organism aging.

## Quiescence, a Motor for Evolution?

One of the first route to multicellularity may have been to aggregate in order to improve resistance to unfavorable environment (see for example Smukalla et al., 2008; Lyons and Kolter, 2015). This kind of cell consortium may have dampened niche fluctuation and created a rudimentary microenvironment that might have launched quiescence independency toward the macroenvironment. This primitive microenvironment may have provided a nutrient buffering effect in which cells, although having not enough resources to divide, could do more than just survive. In this context, we propose that quiescent cells could have launched new functions. At the beginning, these new features were probably not too much demanding in terms of metabolic resources, but some of them might have provided a selective advantage, like the ability to move and thus to explore new environments or escape predation. In fact, in many cases, proliferation and specialization became exclusive. As an example, volvox somatic cells can swim thanks to a flagellum but they have lost the capacity to proliferate. Reciprocally, volvox gonidia cells do not have flagella, but they can divide. This swim or divide specialization echoes the segregation of somatic functions and reproduction into distinct cell types and it thought to be one of the first key steps in the evolution of multicellularity (King, 2004). Furthermore, there are growing evidences that the reverse is true and that rapid cell division favors dedifferentiation (Guo et al., 2014; Matson et al., 2017) and cell transformation (Chen et al., 2019). Thus, we propose that with the emergence of multicellularity, quiescence became no longer confined to a survival form but may have favored cell specialization thereby launching division of labor.

With the complexification of multicellular organisms, proliferation-quiescence transitions detached from the availability in nutrients and became largely independent of the macroenvironment to rely mostly on signals from neighboring cells. Secreted compounds such as metabolites, vesicles or proliferating factors, and importantly, the extracellular matrix, play a crucial role in the control of quiescence, just like physical cues such as the tension induced by the tissue architecture. This has been comprehensively reviewed in Fiore et al. (2018).

The possibility of being quiescent in a plentiful environment could have fostered some specific metabolic activities, as shown for contact-inhibited fibroblasts (Lemons et al., 2010). In fact, in heterotrophs such as humans, a part of quiescent cell metabolism is dedicated to energy production through catabolism, breakdown and re-synthesis of proteins. Part of it can also be devoted to specific processes, such as the synthesis of extra-cellular matrix (ECM) compounds. Therefore, some dedicated metabolic pathways can be active in the total absence of proliferation (Valcourt et al., 2012). This probably opened new avenues for the emergence of cellular functions specific of quiescent cells. One typical example is the primary cilium, a structure with several key sensing functions that is assembled upon quiescence establishment by many types of mammalian cells (Satir et al., 2010). Thus, quiescence and cell specialization could have progressed hand in hand, progressively moving away quiescence from its ancestral dependency toward the macroenvironment. In this scheme, multicellularity might have arisen first, then, microenvironment and quiescence could have co-evolved, reciprocally acting on each other.

**FIGURE 1:**
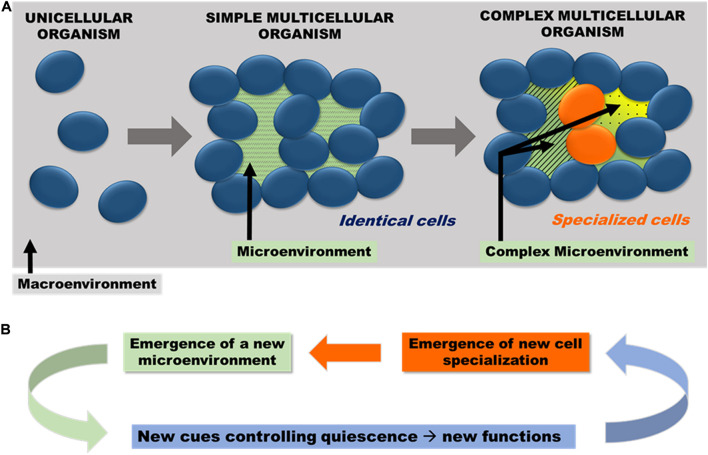
**(A)** Emergence of microenvironment and cell specialization. By “simple multicellular organism,” we mean a multicellular entity composed of identical cells and by “complex multicellular organism,” an organism composed of non-identical cells i.e., cells with different cellular properties. As such, “complex multicellularity” emerged together with cell specialization. Multicellularity physically generates a microenvironment that isolates some cells from the direct contact with macroenvironment. Complex microenvironment (secreted metabolites, diffusive proteins, extracellular matrix, etc.) and the tissue architecture become key in controlling quiescence. Hence, multicellularity may have allowed quiescence to evolve away from nutritional signals. **(B)** Quiescence in a buffered microenvironment favors the emergence of cell specialization, which in turn generates a more complex microenvironment hence closing an auto-complexification loop.

If cell specialization and quiescence co-evolved after the multiple independent emergences of multicellularity, it may be impossible to identify common properties of quiescent cells that would be posterior to multicellularity. However, as discussed above, some ancient quiescence properties inherited from unicellular organisms were conserved. Quiescence, initially selected for its ability to increase survival by slowing down proliferation, would have allowed diversifying cellular functions once reprocessed in a multicellular context. This was made possible because multicellularity eventually isolate some cells from the direct contact with macroenvironment. Hence, through the generation of a microenvironment, multicellularity could have allowed quiescence to evolve away from nutritional signals. This new form of quiescence would have favored the emergence of cell specialization, which in turn would have made the microenvironment more complex, resulting in a sort of relay race between quiescence and multicellularity (Figure 1). This co-evolution between quiescence and cell specialization, by offering major functional and structural opportunities to innovate might have been chief to foster life diversification. Thus, quiescence may have played an important role in multicellular evolution.

## Data Availability Statement

The original contributions presented in the study are included in the article/supplementary material, further inquiries can be directed to the corresponding author.

## Author Contributions

All authors listed have made a substantial, direct and intellectual contribution to the work, and approved it for publication.

## Conflict of Interest

The authors declare that the research was conducted in the absence of any commercial or financial relationships that could be construed as a potential conflict of interest.

## Publisher’s Note

All claims expressed in this article are solely those of the authors and do not necessarily represent those of their affiliated organizations, or those of the publisher, the editors and the reviewers. Any product that may be evaluated in this article, or claim that may be made by its manufacturer, is not guaranteed or endorsed by the publisher.
